# Burden of Visceral Leishmaniasis in Villages of Eastern Gedaref State, Sudan: An Exhaustive Cross-Sectional Survey

**DOI:** 10.1371/journal.pntd.0001872

**Published:** 2012-11-01

**Authors:** Yolanda Kathrin Mueller, Fabienne Nackers, Khalid A. Ahmed, Marleen Boelaert, Jean-Claude Djoumessi, Rahma Eltigani, Himida Ali Gorashi, Omer Hammam, Koert Ritmeijer, Niven Salih, Dagemlidet Worku, Jean-François Etard, François Chappuis

**Affiliations:** 1 Epicentre, Paris, France; 2 Médecins Sans Frontières - Operational Centre Geneva, Geneva, Switzerland; 3 Institute of Tropical Medicine, Antwerp, Belgium; 4 Federal Ministry of Health, Khartoum, Sudan; 5 Community Medicine Department, University of Gedaref, Gedaref, Sudan; 6 Médecins Sans Frontières - Operational Centre Amsterdam, Amsterdam, Holland; 7 Institut de Recherche pour le Développement (IRD), Montpellier, France; 8 Geneva University Hospitals and University of Geneva, Geneva, Switzerland; Institut Pasteur de Tunis, Tunisia

## Abstract

**Background:**

Since December 2009, Médecins Sans Frontières has diagnosed and treated patients with visceral leishmaniasis (VL) in Tabarak Allah Hospital, eastern Gedaref State, one of the main endemic foci of VL in Sudan. A survey was conducted to estimate the VL incidence in villages around Tabarak Allah.

**Methods:**

Between the 5^th^ of May and the 17^th^ of June 2011, we conducted an exhaustive door-to-door survey in 45 villages of Al-Gureisha locality. Deaths were investigated by verbal autopsies. All individuals with (i) fever of at least two weeks, (ii) VL diagnosed and treated in the previous year, and (iii) clinical suspicion of post-kala-azar dermal leishmaniasis (PKDL) were referred to medical teams for case ascertainment. A new case of VL was a clinical suspect with a positive rk39 rapid test or direct agglutination test (DAT).

**Results:**

In the 45 villages screened, 17,702 households were interviewed, for a population of 94,369 inhabitants. The crude mortality rate over the mean recall period of 409 days was 0.13/10'000 people per day. VL was a possible or probable cause for 19% of all deaths. The VL-specific mortality rate was estimated at 0.9/1000 per year.

The medical teams examined 551 individuals referred for a history of fever of at least two weeks. Out of these, 16 were diagnosed with primary VL. The overall incidence of VL over the past year was 7.0/1000 persons per year, or 7.9/1000 per year when deaths possibly or probably due to VL were included. Overall, 12.5% (11,943/95,609) of the population reported a past VL treatment episode.

**Discussion and Conclusion:**

VL represents a significant health burden in eastern Gedaref State. Active VL case detection had a very low yield in this specific setting with adequate access to care and may not be the priority intervention to enhance control in similar contexts.

## Author Summary

Visceral leishmaniasis (VL) is a life-threatening parasitic disease, transmitted by a sandfly. A survey was conducted to estimate the VL incidence in 45 villages located in the eastern part of Gedaref State, the main endemic focus of VL in Sudan. Between the 5^th^ of May and the 17^th^ of June 2011, we interviewed 17,702 households for a population of 94,369.

Sixteen individuals were diagnosed with primary VL through active case-detection, and 725 reported VL treatment over the past year. The overall incidence rate of VL over the past year was 7.0/1000 persons per year. The crude mortality rate over the mean recall period of 409 days was 0.13/10'000 persons per day. VL was a possible or probable cause for 19% of all deaths. Taking also into account the VL-specific mortality of 0.9/1000 per year, the incidence was estimated at 7.9/1000 per year. Overall, 12.5% of the population reported having been treated for VL in the past.

VL is a major public health issue in Gedaref. Active VL case detection had a very low yield in a context of adequate access to care. Such strategy seems redundant if patients already have access to care.

## Introduction

Visceral leishmaniasis (VL), also called kala-azar, is a parasitic disease caused by members of the *Leishmania donovani complex* (*L. donovani* and *L. infantum*) and transmitted by the female phlebotomine sand flies of the genera *Phlebotomus* (Old World) and *Lutzomyia* (New World). It mainly affects areas in South Asia (India, Bangladesh, and Nepal) and Eastern Africa, where Sudan is the most affected country, followed by Ethiopia, Kenya, Somalia and Uganda. In Sudan, the causative agent is *L. donovani*, transmitted by the *Ph. orientalis*.

Gedaref State is the main endemic area of VL in Sudan. Passive detection figures from 1996 to 1999 have shown a mean yearly incidence between 6.6 and 8.4 VL cases per 1000 persons, with a large variation between villages (from 0 to 60 cases per 1000 persons per year) [1], [2]. Villages with high incidence are clustered along two rivers (Atbarah and Rahad), in areas of low altitude and high rainfall. Leishmanin skin testing, a marker of past exposure to the disease, has been shown to be positive in 21.6% of the population of the Atbarah area [3].

Many individuals infected by *L. donovani* have subclinical infection, while others develop clinical VL, a devastating illness that is usually fatal when left untreated. In Sudan, clinical signs develop gradually 2 weeks to 1 year after infection (in most cases after 2 to 4 months). Typical features are persistent fever, splenomegaly, weight loss and lymphadenopathies [4]. Post-kala-azar dermal leishmaniasis (PKDL) is a skin rash appearing after VL treatment, affecting up to 50% of treated cases in Sudan [5]. PKDL usually appears within 6 months after apparent cure and can last for months or years. *Leishmania* parasites can be found in smears of the skin lesions, and PKDL lesions are suspected to be an important parasite reservoir for human-to-human transmission. In Sudan, most lesions heal spontaneously. If not, PKDL treatment is challenging [6].

Some vector-control strategies such as indoor residual spraying have been shown to reduce the density of sand fly vectors in the Indian subcontinent [7], where the vector *Ph. argentipes* exhibits a behaviour different from the Sudanese vector. In Africa, bednet use has been shown to potentially reduce VL incidence [8]. Also, strategies to promote early detection and treatment have been shown to reduce case-fatality rates of VL in Brazil, where zoonotic VL is caused by *L. infantum (syn. L. chagasi)* [9]. Furthermore, early detection and treatment of anthroponotic VL patients is also believed to lower transmission through the reduction of the human reservoir [10]. This is supported by one pilot study that achieved good results using a combination of active detection, treatment and indoor residual spraying after a local outbreak of VL in one village located in the Bihar State of India [11]. However, although recommended, this strategy has never been formally evaluated in *L. donovani* endemic areas and is rarely implemented.

Since December 2009, Médecins Sans Frontières (MSF) has been diagnosing and treating patients presenting at Tabarak Allah Hospital, located in Al-Gureisha locality of the the Atbarah focus. MSF intended to conduct a cluster-randomized trial to evaluate the impact of an active VL and PKDL case detection strategy on the incidence of clinical VL. For the appropriate planning of this trial, a baseline survey was conducted in eligible villages around Tabarak Allah Hospital. The main objective of this survey was to estimate the incidence rate of VL over a one-year period at the village level. Additionally, we also aimed at retrospectively estimating the crude and VL-specific mortality rates, the proportion of VL cases missed by the passive case detection system in place, the proportion of the population treated for VL in the past, and the proportion of PKDL among patients previously treated for VL.

## Methods

### Ethics Statement

Ethical clearance was granted from the Sudanese National Ministry of Health's Research Ethics Review Committee. Written authorization to conduct the study was obtained from the Gedaref Ministry of Health and each head of village. Each head of household provided oral informed consent to the collection of demographical data, history of VL treatment, skin rash after treatment, and presence of fever of at least two weeks among household members. A referral form was given for each individual presenting with fever of more than two weeks, with suspicion of PKDL or having been treated for VL in the last year. The information included in these forms was not identifying and individuals were free to reach or not the medical team for clinical investigation. An additional oral consent was obtained from clinical suspects before testing for VL. The choice of oral consent was made because of the low literacy rate in the study area and the unlikelihood to easily find an impartial literate witness for each household. The Sudanese National Ministry of Health's Research Ethics Review Committee expressly approved the method of oral consent without use of a witness or written record of oral consent.

Between the 5^th^ of May and the 17^th^ of June 2011, we conducted an exhaustive door to door survey in the 45 villages of Al-Gureisha locality, covering a population of about 85,000 inhabitants. The survey villages were grouped into four geographical areas. Each area was surveyed by four field teams and one medical team.

Demographic information (age, sex, household composition on the day of survey and one year prior, number of births, deaths and movements within the past year) was collected by the field teams in each household. A household was defined as all people living together under the responsibility of one head of household and eating regularly together. For each household member, the history of VL treatment and possible subsequent PKDL was also recorded. The number and the causes of any death occurring in the past year were investigated in order to identify deaths possibly attributable to VL. Verbal autopsies were conducted for all reported deaths except for neonatal, delivery-related, and accidental deaths, as these were unlikely to be related to VL. Maternal deaths not directly related to delivery were investigated, as VL during pregnancy is known to be associated with increased treatment toxicity and mortality [12], [13].

Individuals with fever of at least two weeks duration, individuals diagnosed and treated for VL during the past year, and clinical suspects of either PKDL or VL relapse (independently of the time elapsed since treatment) were referred to the medical teams for clinical examination and case ascertainment. New clinical VL suspects (defined as fever for at least two weeks with at least one of the following: splenomegaly, lymphadenopathies or history of weight loss) were tested with an rK39 antigen-based rapid test (DiaMed IT-Leish) [14] and, if negative, with the direct agglutination test (DAT) [15], [16] for VL confirmation. A new VL case was defined as a clinical suspect who was confirmed either by the rK39 or the DAT. New VL cases, suspected VL relapses, and moderate and severe PKDL cases were referred to Tabarak Allah Hospital. Because of the self-healing nature of PKDL in Sudan and the potential toxicity of the recommended SSG treatment, mild PKDL cases were not offered SSG treatment [6] and therefore were not referred to Tabarak Allah Hospital.

To estimate the incidence rates at the village level, the population was exhaustively screened. We calculated a sample size of 266 deaths to estimate a proportion of deaths due to VL of 30% with a 5% precision (alpha 0.05). Based on an expected total number of deaths around 1500 (corresponding to an annual mortality rate of 0.5/10'000 persons per day), we planned to investigate the cause of every fifth death through verbal autopsy, using a systematic sampling procedure. All deaths were recorded consecutively on a tally sheet, with the death to be investigated pre-highlighted. As the data collected during the first three weeks of the survey showed a number of deaths much lower than expected, we later conducted verbal autopsies for every reported death. The analysis of the causes of death was weighted accordingly.

All verbal autopsies were reviewed independently by two clinicians experienced in VL and fluent in Arabic. In case of disagreement, the files were reviewed by a third expert clinician, with the help of a translator, and his verdict was final. Death was considered possibly due to VL if the respondent mentioned fever of at least two weeks duration and either one of the following: enlarged lymph nodes, a visible mass in the left upper part of the abdomen (spleen side), or weight loss, during the final illness of the deceased. Death was considered as probably due to VL if it occurred during treatment for VL (clearly mentioned by the relatives of the deceased) in a treatment facility offering reliable VL diagnosis (i.e. rk39 rapid test, DAT or microscopic examination of lymph node aspirate with quality control in place). If a death was reported to have occurred in another treatment facility during VL treatment, it was considered as possibly due to VL.

The event chosen to define the start of the recall period (covering the past year) was the presidential elections in Sudan, which occurred on the 10^th^ and 11^th^ of April 2010. The average recall period (referred hereafter as the “the past year”) was therefore 409 days. The end of the sesame harvest (end of October 2010) was used to define a 6-month recall period. VL incidence rate over the period was calculated by summing the new VL cases detected during the survey, the VL cases and the deaths possibly/probably due to VL reported over the recall period, divided by the mid-year population.

All documents were translated in Arabic and back-translated into English, and were subjected to pilot testing with subsequent update before the start of the survey. Data were entered in the EpiData software (EpiData, Odense, Denmark) by four data entry clerks. Data were analysed using the Stata 11 software (Stata Corporation, College Station, Texas, USA). Description of geographical information was performed using the QuantumGIS software, version 1.7.0. The coordinates of the Atbarah River were obtained by manually drawing along the river in Google Earth.

## Results

A total of forty-five villages were screened, corresponding to 17,965 households, 17,702 (98.5%) of which gave verbal consent to participate. The mid-year population was 94,369 inhabitants. The median household size at the time of the survey was 5 persons (interquartile range (IQR) 3 to 7 persons). The male/female sex ratio was 1.08. The median age was 15 years (IQR 7 to 30 years). The median population size by village was 1241 inhabitants (IQR 692 to 3113) at the time of the survey.

Overall, 12.5% (11,943/95,609) of the population reported having been treated for VL in the past, varying between 1.8% and 34.7% across villages. The medical teams assessed 725 individuals reporting VL treatment in the past year. Out of them, 125 (24%) mentioned a rash occurring within a median of 2 months after VL treatment (IQR 1 to 4 months) and lasting for a median of 3 months (IQR 1 to 10 months). Overall, PKDL was diagnosed in 260 cases (123 treated within the past year, 137 treated more than one year ago), corresponding to 0.3% of the survey population. The prevalence of PKDL cases ranged from 0 to 1.5% across villages. Most of the observed PKDL rashes were mild (81.5%) and none required treatment. In addition, the medical teams referred 40 patients for suspected VL relapse. Microscopic examination of lymph node aspirate was negative in 38 individuals and positive in 2 patients therefore diagnosed with VL relapse and treated.

**Figure 1:**
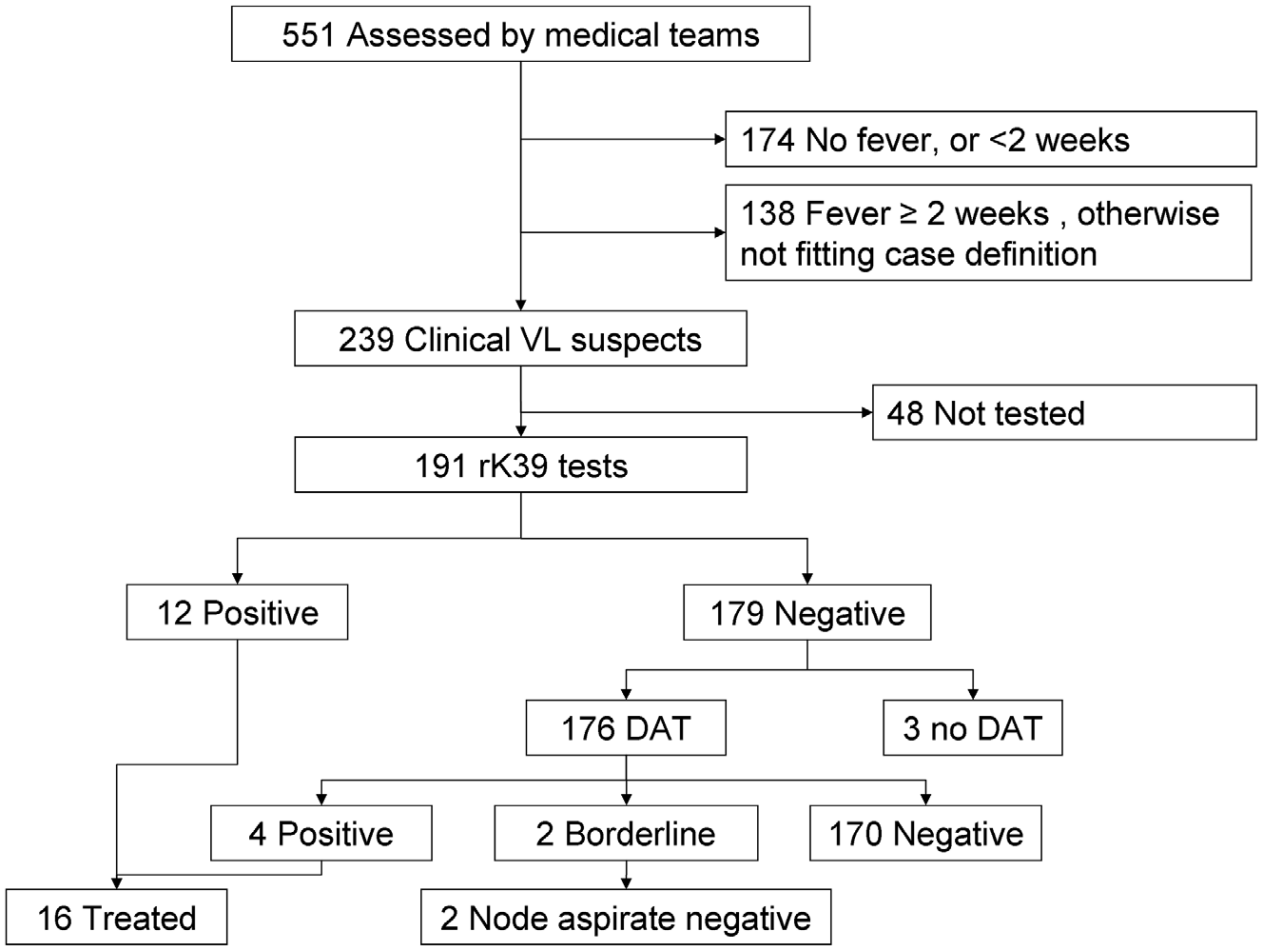
Flowchart of individuals assessed for fever of at least 2 weeks duration, eastern Gedaref State, Sudan, May-June 2011. * Positive rK39 test in PKDL suspect, never treated for VL in the past. DAT: Direct Agglutination Test.

**Figure 2:**
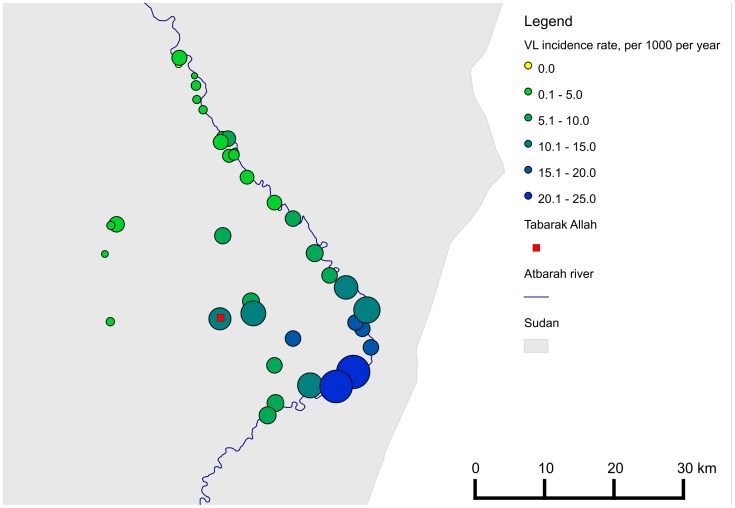
Incidence of VL over past year, eastern Gedaref State, Sudan, May-June 2011. * The size and the colour of the circles are proportionate to VL incidence.

The medical teams examined 551 subjects not previously treated for VL (Figure 1). Of these, 239 qualified as new clinical VL suspects, while the remaining 312 did not meet the case definition. Sixteen patients were ultimately diagnosed with primary VL (12 by rK39 rapid test and 4 by DAT), 85% of whom had actually sought care previously. Compared to the 725 cases treated in the past year, the active case detection therefore allowed to diagnose 16 (2%) additional of new cases. The age and sex distribution of the 741 VL cases newly diagnosed or treated in the past year is shown in Table 1. Males represented 54% of the cases, and 59.5% of the cases were aged from 5 to 14 years. The overall incidence rate of VL cases over the mean recall period of 409 days was 7.0/1000 persons per year. VL incidence rates by village varied between 0 and 23.0/1000 persons per year (Figure 2).

**Table 1:** Age and sex distribution of VL cases in the past year (725 previously treated cases and 16 new cases) and of the general population, eastern Gedaref State, Sudan, May-June 2011.

	VL cases			General population		
	Male	Female	Total	Male	Female	Total*
Age group	n (%)	n (%)	n (%)	n (%)	n (%)	n (%)
- 0 to 4 years	67 (16.6)	63 (18.7)	130 (17.5)	8620 (17.4)	8017 (17.4)	16667 (17.4)
- 5 to 14 years	250 (61.9)	191 (56.7)	441 (59.5)	14613 (29.5)	14013 (30.5)	28644 (30.0)
- 15 and above	82 (20.3)	78 (23.1)	160 (21.6)	26254 (53.0)	23919 (52.0)	50220 (52.5)
- Missing	5 (1.2)	5 (1.5)	10 (1.3)	34 (0.1)	40 (0.1)	78 (0.1)
Total	404 (54.5)	337 (45.5)	741 (100.0)	49521 (51.8)	45989 (48.1)	95609 (100.0)

* Missing sex in general population: 99 (30 aged 0 to 4 years old, 18 aged 5 to 14 years old, 47 aged 15 and above, 4 with missing age).

Five hundred and six deaths were reported, resulting in a crude mortality rate (CMR) of 0.13/10,000 persons per day. At the village level, the CMR varied between 0.02 and 0.30/10,000 per day, with a median of 0.14/10,000 per day. Accidental deaths represented 19.6% of all deaths, while neonatal and delivery-related deaths represented 14.8% and 1.8%, respectively. The remaining deaths were investigated by 171 verbal autopsies, corresponding to 299 deaths (32 sampled in the initial period of the survey given a weight of five, plus 139 for the remaining time of the survey). Taking into account the weighting, VL was a possible or probable cause of death in respectively 3.7% and 26.1% of verbal autopsies, or 2.4 and 16.6% when extrapolated to the total number of deaths. Other main causes of death were acute febrile illnesses (17.1%) and death related to chronic non-communicable diseases, mainly cardiovascular disease and diabetes (9.6%). Among the deaths probably/possibly due to VL (weighted n = 89), 45% occurred at home, 89% had received a medical treatment, and 53% had a clear history of VL treatment. The VL-specific mortality rate was 0.9/1000 persons per year. Taking into account these deaths possibly or probably due to VL, the overall incidence rate of VL cases over the recall period would reach 7.9/1000 per year.

## Discussion

In eastern Gedaref State, one out of 127 inhabitants was affected by VL over the past year. These incidence rates were lower than figures previously reported from the same region [1]. Still, a large proportion of the population (12.5%) has been affected by clinical VL in the past, reaching over one third in some villages. Also, one fifth of all deaths in the previous year may have been due to VL. However, there was no clear correlation between VL incidence and crude mortality rates at village level, which were overall lower than the reported average in Sudan [17], [18]. Still, VL represents an important burden in these communities, even between peaks of high incidence that occur approximately every six to ten years in Sudan [2], [18].

Interestingly, although gender is usually mentioned as a risk factor for VL [19], our data did not show a strong male predominance among cases compared to the general population. Project data from Tabarak Allah Hospital report 55% males among patients treated for VL, which is lower than figures reported from other treatment centres of Gedaref in the past [20], [21]. It is unclear whether this difference reflects differential access issues, changing epidemiology over time, or focal differences in transmission patterns. By contrast, age was clearly associated with VL: almost 60% of the cases were aged between five and 14 years, while this age group only represented 30% of the general population.

Up to one quarter of patients treated for VL within the past year reported some skin change consistent with PKDL, appearing within a median of two months after treatment and lasting for a median of three months. This is lower than reported in previous studies where up to 50% of treated VL patients developed PKDL [22], [23]. However, we only reported the proportion of PKDL among patients treated within the past year. Some patients were therefore likely to develop PKDL within the year after completion of the survey. Also, patients may not have reported mild and short-lasting PKDL. Although most PKDL cases are mild, they could still represent a reservoir of parasites, as *L. donovani* parasites can be detected in skin lesions [24]. None of the PKDL treatment currently available appears appropriate to treat mild cases, either because of toxicity (antimonials, conventional amphotericin B), teratogenicity (miltefosine) or cost (liposomal amphotericin B). As long as there is no definite evidence for the role of PKDL cases in the transmission chain, it is difficult to advocate for the development of better and simpler treatments for this condition.

Active VL case detection allowed us to detect an additional two percent of cases (n = 16/725). This appears as a very low yield for such a labour-intensive and costly operation. The survey was conducted at a time of the year when the number of new cases recorded at Tabarak Allah hospital is usually low. Thus, our results confirm that the incidence of clinical VL is low in May and June, and that this is not related to restricted access or use of health services. Active case detection may have detected more cases if it had been done from September to November, just after the rainy season, when incidence is believed to be higher and when many clinical cases have not yet sought medical care. However, the 2010–2011 Tabarak Allah hospital data neither show a clear seasonal trend, nor a large seasonal difference in delays for seeking care. Adequate access to care was confirmed by the short duration of symptoms reported by most VL cases on admission to Tabarak Allah Hospital (source: MSF project data). Also, most of the 16 new VL cases detected by the survey teams had actually previously sought care at health centres but were not adequately diagnosed with VL during that consultation. Our results show that when good-quality services are made accessible to a population that is well sensitized, active case detection might not be relevant. Based on our results, MSF decided not to proceed with the initially planned trial on active case detection, and not to recommend active case detection as a control strategy in the area.

A recent mathematical transmission model based on south Asian data suggested that VL treatment only had almost no effect on the overall intensity of transmission, which was mainly attributed to asymptomatically infected hosts [22], [23], [25]. These results cannot be extrapolated directly to Sudan where the VL epidemiology is very different, especially regarding the asymptomatic to symptomatic ratio that is much lower than in India [26]. Nevertheless it would be useful to adapt this model with Sudanese data, in order to guide efforts for VL control in the future.

The main limitation of our survey was the length of the recall period (over one year). Memory inaccuracies may have led to an overestimation of VL incidence, since VL cases that occurred more than one year prior the survey may have been reported as occurring within the past year. Some VL relapses treated during the past year may have been erroneously counted as new VL cases. Seasonal workers who left the area and developed VL later and elsewhere in Sudan were not included in the incidence results. These potential biases acted in opposite directions, which may have mitigated their impact on the estimated incidence of VL. Moreover, these biases being similar across villages, the relative differences in VL incidence still identify the villages most affected by VL in the study area.

The one out of five sampling of deaths submitted to verbal autopsy in the initial period of the survey may have caused some selection of the deaths investigated, which could have led to an overestimation of the proportion of deaths attributed to VL. However, the characteristics of deaths and the proportion attributable to VL were similar between the two periods, indicating no such phenomenon. We cannot exclude that some of the deaths attributed to VL may have been due to another disease causing similar symptoms, such as tuberculosis, advanced HIV infection, or cancer.

## Conclusions

VL represents a significant health burden in the villages of eastern Gedaref State. The disease was among the major causes of death in the area. Active VL case detection through door-to door screening did not prove to be an efficient way to diagnose new VL cases likely due to current good access to VL care and relatively low prevalence of cases because the survey took place during a low-transmission period.

## Supporting Information

Checklist S1 STROBE checklist.
(PDF)
